# Oral Drug Absorption and Drug Disposition in Critically Ill Cardiac Patients

**DOI:** 10.3390/pharmaceutics15112598

**Published:** 2023-11-07

**Authors:** Lars-Olav Harnisch, Jürgen Brockmöller, Anne Hapke, Juliane Sindern, Ellen Bruns, Ruben Evertz, Karl Toischer, Bernhard C. Danner, Dorothee Mielke, Veit Rohde, Tammam Abboud

**Affiliations:** 1 Department of Anesthesiology, University of Göttingen Medical Center, 37075 Göttingen, Germany; 2 Department of Clinical Pharmacology, University of Göttingen Medical Center, 37075 Göttingen, Germany; juergen.brockmoeller@med.uni-goettingen.de (J.B.); ebruns@med.uni-goettingen.de (E.B.); 3 Department of Neurosurgery, University of Göttingen Medical Center, 37075 Göttingen, Germany; ahapke@ukaachen.de (A.H.); dorothee.mielke@med.uni-goettingen.de (D.M.); veit.rohde@med.uni-goettingen.de (V.R.); tammam.abboud@med.uni-goettingen.de (T.A.); 4 Department of Otorhinolaryngology-Head and Neck Surgery, RWTH Aachen University Hospital, 52074 Aachen, Germany; 5 Department of Anesthesiology and Critical Care Medicine, Medical Center, University of Freiburg, 79106 Freiburg, Germany; 6 Department of Cardiology and Pneumology, University of Göttingen Medical Center, 37075 Göttingen, Germany; ruben.evertz@med.uni-goettingen.de (R.E.); ktoischer@med.uni-goettingen.de (K.T.); 7 Department of Cardiac, Thoracic and Vascular Surgery, University of Göttingen Medical Center, 37075 Göttingen, Germany; bernd.danner@med.uni-goettingen.de

**Keywords:** esomeprazole, intensive care medication, cardiogenic shock, enteral medication

## Abstract

(1) Background: In critically ill cardiac patients, parenteral and enteral food and drug administration routes may be used. However, it is not well known how drug absorption and metabolism are altered in this group of adult patients. Here, we analyze drug absorption and metabolism in patients after cardiogenic shock using the pharmacokinetics of therapeutically indicated esomeprazole. (2) Methods: The pharmacokinetics of esomeprazole were analyzed in a consecutive series of patients with cardiogenic shock and controls before and after elective cardiac surgery. Esomeprazole was administered orally or with a nasogastric tube and once as an intravenous infusion. (3) Results: The maximum plasma concentration and AUC of esomeprazole were, on average, only half in critically ill patients compared with controls (*p* < 0.005) and remained lower even seven days later. Interestingly, esomeprazole absorption was also markedly compromised on day 1 after elective surgery. The metabolites of esomeprazole showed a high variability between patients. The esomeprazole sulfone/esomeprazole ratio reflecting CYP3A4 activity was significantly lower in critically ill patients even up to day 7, and this ratio was negatively correlated with CRP values (*p* = 0.002). The 5′-OH-esomeprazole and 5-O-desmethyl-esomeprazol ratios reflecting CYP2C19 activity did not differ significantly between critically ill and control patients. (4) Conclusions: Gastrointestinal drug absorption can be significantly reduced in critically ill cardiac patients compared with elective patients with stable cardiovascular disease. The decrease in bioavailability indicates that, under these conditions, any vital medication should be administered intravenously to maintain high levels of medications. After shock, hepatic metabolism via the CYP3A4 enzyme may be reduced.

## 1. Introduction

In critically ill patients, the parenteral and enteral routes may be used for feeding and drug administration. Early enteral nutrition has been shown to reduce mortality in critically ill patients [1,2,3]. Although the enteral route is also suggested for drug administration [1,4,5,6], many critically ill patients will receive medication parenterally to ensure that the intended dose is administered with 100% bioavailability. To adequately use the enteral route, a gastrointestinal tract capable of motility and absorptive function is a prerequisite [7]. However, the enteral route of drug administration in critically ill patients is altered by many different mechanisms; therefore, serum drug levels were low in critically ill patients after oral administration [8,9,10,11,12,13,14,15].

Cardiogenic or septic shock can lead to multiple organ failure, life-threatening complications, and increased mortality. Organ insufficiency, less commonly considered in shock conditions, involves the gastrointestinal tract, for which a marked reduction in motility has been demonstrated [16,17,18,19]. Shock can also alter the pharmacokinetics of drugs by affecting metabolic organ function, blood flow, drug distribution, metabolism, and excretion.

We investigated the absorption and metabolism of enterally administered esomeprazole compared to intravenously administered esomeprazole in patients treated for cardiogenic shock compared to elective cardiac surgical patients before and after surgery, as there is a lack of valid knowledge on this issue [20].

Proton pump inhibitors (PPIs) are subject to a complex pharmacokinetic process that involves jejunal absorption and transport through the blood to parietal cells in the stomach, where PPIs are converted under highly acidic conditions to reactive cyclic sulfenamides that form disulfide bonds with SH groups of the proton pump (a H^+^ K^+^-ATPase), resulting in irreversible inactivation. PPIs are rapidly eliminated from systemic circulation by the liver cytochrome P450 enzymes CYP2C19 and CYP3A4 [21]. Due to their almost ubiquitous application in intensive care medicine, measuring blood concentrations of proton pump inhibitors may be a suitable means to study changes in drug absorption and metabolism in intensive care medicine.

We hypothesized that enterally administered esomeprazole absorption may be altered in shock patients compared with patients with stable cardiovascular systems. Furthermore, we investigated the metabolites of esomeprazole and hypothesized that hepatic metabolism by the cytochrome P450 enzymes 2C19 and 3A4 would also be altered in shock patients compared with postoperative patients. 5′-OH-esomeprazole and 5-O-desmethylesomeprazole metabolites are generated almost exclusively by CYP2C19, while CYP3A4 generates esomeprazole sulfone. As introduced, it would be best to administer drugs and foods in intensive care by oral/intestinal route as soon as possible. However, we must know how well the drugs are absorbed during shock and the following days. In this context, esomeprazole may serve as a probe drug. Its pharmacokinetics might provide general information on alterations in drug absorption and metabolic elimination relevant to a wide range of drugs.

## 2. Materials and Methods

This study was approved by the University Research Ethics Board, and written informed consent was obtained prior to the first study-related action of all subjects or their legal representatives. This study was carried out at a university hospital in Germany certified as a national cardiac arrest center.

We conducted a prospective, nonrandomized, and open (i.e., nonblind) clinical pharmacokinetic study. Patients were eligible if they were in cardiogenic or combined cardiogenic–septic shock (defined as a requirement for catecholamines despite adequate volume administration and pathological lactate concentrations at the beginning of the study [22]), between 18 and 80 years of age and received a nasogastric tube for clinical reasons. Patients who underwent elective cardiac surgery served as a control group. Patients were not eligible if they took clopidogrel or suffered from chronic bowel disease or benzodiazepine abuse. In all participants, treatment with a proton pump inhibitor was clinically indicated.

The multi-unit pellet system (Nexium^®^ mups^®^, Grünenthal GmbH, Aachen, Germany) was used for oral dosing because it can be administered orally and through a nasogastric tube. For intravenous dosing, an infusion of Nexium^®^ 40 mg (Grünenthal, Aachen, Germany) was prepared according to the manufacturer’s instructions and applied as a short infusion for 10 min. For both routes of application, the dose was 40 mg of esomeprazole. Patients were included within 72 h of the triggering event leading to cardiogenic shock, respectively, the day before elective surgery.

Esomeprazole was administered daily per os or by a nasogastric tube; only on day 4, it was administered intravenously in all subjects. Application of esomeprazole via the nasogastric tube was performed as described by the manufacturer in the summary of product characteristics. The tablets were dissolved in 25 mL tap water in a syringe and gently shaken for 2 min. Then, portions of about 8 mL were applied into the gastric tube, and the syringe was shaken between the portions to reduce sedimentation of the pellets. Finally, the syringe and gastric tube were rinsed with an additional 25 mL of tap water to flush any remaining micropellets into the gastric volume. It was shown earlier that application in this mode results in the same bioavailability compared to swallowing the corresponding tablets [23]. The first blood sample was taken before the first dose on the respective study day (“trough”, “baseline”); additional blood samples were taken 30, 60, 90, 120, 180, 240, 360, and 480 min after drug administration (S-Monovette^®^ potassium ethylene diamine tetra-acetic acid as anticoagulant, 2.6 mL, Sarstedt, Nürnbrecht, Germany); we studied on days 1, 3, 4, and 7 after the day of hospital administration in the group after shock and on days 1, 3, and 4 in the control group undergoing elective cardiovascular surgery. On day 4, in both groups, esomeprazole was administered intravenously. After blood collection, the samples were centrifuged at room temperature within 2 h after drawing for 10 min at 2000 g, and the plasma supernatant was stored at −20 °C until further handling. Final analysis of esomeprazole (S-enantiomer of omeprazole) and its three main metabolites, 5′-hydroxy-esomeprazole (OH), 5-O-desmethyl-esomeprazole (DM), and omeprazole sulfone (SO), was performed in a GLP conform manner using high-pressure liquid chromatography (reverse-phase HPLC column Brownlee SPP RP-Amide, Perkin Elmer, Waltham, MA, USA) combined with tandem mass spectrometry (API 4000^TM^ LC-MS/MS system, Sciex, Framingham, MA, USA). A pharmacokinetic analysis was performed using non-compartmental and compartmental methods (WinNonlin, version 6.4., Princeton, NJ, USA). The parameters of primary interest were Tmax, as the time of maximum blood concentration (Cmax), which characterizes intestinal absorption; lag-time (absorption delay time), as the time between oral dosing and the first increase in blood concentration; and the area under the concentration–time curve (AUC), which is a composite parameter for drug absorption and excretion and corresponds to the proportion of the drug available for action. Bioavailability was calculated as F = (AUC (po)/(AUC (iv)).

Furthermore, we extracted the following clinical data from the charts to investigate their influence on absorption and metabolism. Serum lactate, neuroprotective cooling (qualitatively), cardiac catheterization (qualitatively), left ventricular ejection fraction, creatine kinase, high-sensitive Troponin I (hsTnI), age, body weight, body mass index (BMI), c-reactive protein, duration of surgery, NYHA classification, and ASA classification.

We calculated values for a minimum of 11 patients per group based on the assumption that Tmax and AUC differed by a factor of 2 between the groups (significance level at *p* = 0.05) for a statistical power of 0.8. A Tmax value of 2.1 h with a standard deviation of 0.9 h was assumed for elderly patients, according to the published literature [24,25]. 

Statistical analysis: Data were tested for normal distribution using the Shapiro–Wilk test. Differences between two groups were tested using the Mann–Whitney U test and between more than two groups using the Kruskall–Wallis test, respectively. If a trend was expected, the nonparametric Jonckheere–Terpstra test was used. Differences between days were tested using the Wilcoxon test; corresponding to the mostly descriptive nature of our study, we did not adjust for multiple testing. Data are presented as median and range. All statistical analyses and the boxplots in Figure 2 were performed with IBM SPSS Statistics version 28.0 (Armonk, NY, USA).

## 3. Results

A total of 25 patients were included in this study. The elective/control cohort included 11 patients, and the cardiogenic shock cohort included 14 patients; the cohorts did not differ significantly with respect to their basic demographic characteristics (Table 1). Based on a cut-off point of 1.2 mg/dL for serum bilirubin, three patients in the cardiogenic shock cohort and one patient in the control cohort had moderately reduced liver function. Patients in the study cohort were admitted to the ICU for cardiogenic shock, as currently defined [22]. Patients in the elective cohort were admitted for elective cardiac surgery.

In the study cohort, two patients died before the last study day and one patient died after the first study day. Data collected from these patients up to the time of death were included in the analysis. In the study cohort, 10 patients were resuscitated prior to inclusion in the study. In the elective cohort, four patients underwent coronary artery bypass grafting (CABG), seven patients underwent valve replacement (mitral: four, combination of valve replacement procedures: two), and one patient received combined CABG and valve replacement (aortic).

Table 2 shows the medications that the patients took in addition to esomeprazole on at least one of the trial days; medications were only reported if at least five patients in the respective group received a given medication (Table 2).

**Figure 1:**
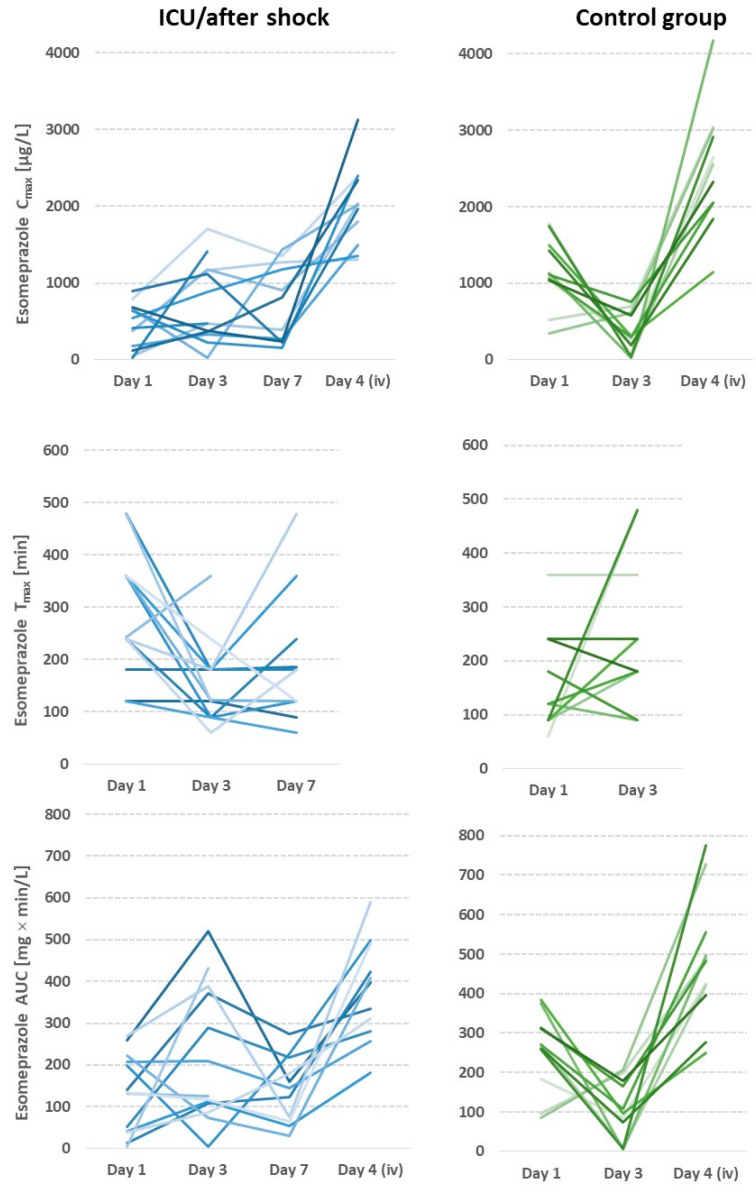
Intraindividual course of esomeprazole pharmacokinetic parameters: left ICU cohort (shades of blue); right elective surgery cohort (shades of green); shades represent individual patients.

Cmax, Tmax, and AUC were statistically significantly different between the ICU and control cohorts (CNTR) on day 1 after hospital admission (Cmax *p* = 0.001, Tmax *p* = 0.005, AUC *p* = 0.005). On days 3, 4, and 7, similar trends as those seen on day 1 were found for all parameters (Table 3). However, no statistically significant results were obtained due to the high individual dispersion of the values; the high individual dispersion is illustrated in Figure 1.

After oral dosing, up to 100-fold differences were found between plasma maximum esomeprazole concentrations with a median (range) of 592.52 (19.24–1774.5) for all patients studied. The ratios of the highest to lowest plasma concentration on day 1 in the ICU cohort were 46.3 compared with 5.1 in the control cohort. We also found substantial interindividual variability in Tmax, Tlag and with median (range) values of 180 (60–480) min; 29.8 (0–81.3), and 152.68 (4.52–519.91), respectively.

**Figure 2:**
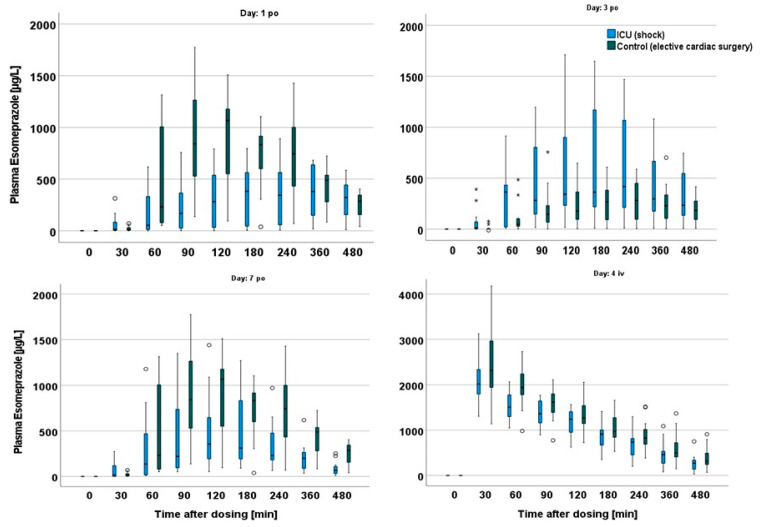
Differences in Cmax between cohorts on the different days studied; outliers are marked by open circles and extreme outliers by asterisks.

On day 1, the variation after oral administration was lower in the control cohort. In particular, there was a significant decrease in AUC and maximum plasma concentration on day 3, the day after the planned intervention, probably explained by significantly reduced cardiac output and opioid analgesia. Interpatient variability was not only related to intestinal absorption but possibly also to liver biotransformation through CYP2C19 and CYP3A4, since high variability was also found after intravenous dosing. The comparison between day 7 of the study group and the first study day of the elective group indicates that AUC was still significantly reduced in the ICU group (elective 271 mg × in/L (85.5–384) vs. ICU 136 mg × min/L (4.5–269), *p* = 0.008) (Figure 2, Table 3).

The absorption delay time (also known as lagtime, Tlag) was similar in both cohorts, so there were no statistically significant differences between the ICU and control cohorts. Tlag is by definition the first appearance of the drug in the blood after enteral dosing. However, that does not reflect the very low blood esomeprazole concentrations up to 90 min after dosing in more than 50% of the patients on days 1 and 2 (Figure 2).

Bioavailability did not differ between the groups on any of the days studied after enteral administration. The only statistically significant difference found was in the elective cohort between day 1 and day 3 (56% (11.7–94) vs. 26.4% (0.9–45.5), *p* = 0.013). However, since metabolite measurements indicated impairment in hepatic elimination via CYP3A enzymes, bioavailability is not easy to interpret in the present study. Impaired absorption results in lower AUC and indicates low bioavailability and impaired hepatic metabolism, which in turn leads to higher AUC and bioavailability.

### Pharmacokinetics of Esomeprazole Metabolites

In the ICU cohort, the AUC of omeprazole sulfone (SO) was higher than the AUC of the other two metabolites in all but one patient and at all time points studied, the AUC of 5-O-desmethyl esomeprazole (DM) was always the smallest. Compared with the other days of oral esomeprazole dosing, the AUC of SO was lowest on day 1 with the exception of the aforementioned patient. However, this outlier patient generally had impaired liver function, as indicated by a serum bilirubin concentration of 2.2 mg/dL. On the second day of this study, the highest AUC values for SO were found in 12 out of 14 patients in the ICU cohort, with only two individual exceptions where the AUC of 5′-OH esomeprazole exceeded that of sulfone. In three patients, the AUCs of OH and DM increased on day 2 compared with day 1. A higher AUC of DM was measured in three different patients compared with day 1, and OH in one patient.

The differences between cohorts were only statistically significant for SO on day 1 (elective 198 mg × min/L (45.5–379) vs. ICU 41 mg × min/L (5.1–287), *p* = 0.001). On day 3 of the pharmacokinetic analysis, which corresponds to the postoperative day in the elective cohort, the highest metabolic ratio values were obtained for most patients, especially those of OH and DM, reflecting the activity of CYP2C19. The SO/ESOM ratios reflecting CYP3A4 activity reached higher values in the elective cohort than in the ICU cohort.

**Figure 3:**
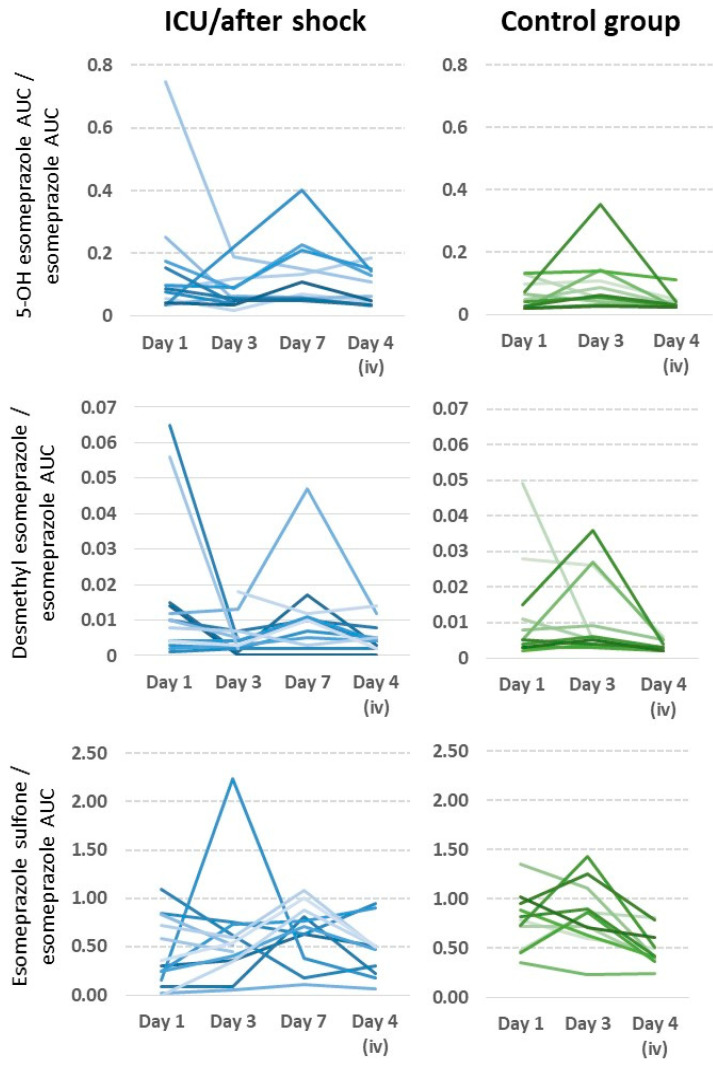
Intra- and interindividual changes in CYP2C19 and CYP3A4 activity in ICU patients after shock (shades of blue) compared to patients undergoing elective cardiac surgery (shades of green); shades represent individual patients. Although there is significant scatter mainly caused by very low concentrations of the parent drug, the quite small ratios of esomeprazole sulfone/esomeprazole on days 1 and 3 are apparent in about 50% of patients after shock, and it is also apparent that these ratios recovered primarily on day 7. Low metabolic ratios after intravenous dosing are explained by the lack of first-pass metabolism. Furthermore, biotransformation in the elective surgery group was not compromised the day after cardiac surgery, although intestinal absorption and bioavailability were significantly compromised in these patients on that day.

The ratios, normalized for differences in bioavailability, supported the same conclusions as the AUCs of the metabolites: statistically significant differences were found for SO/ESOM on day 1 (elective 0.79 mg × min/L (0.35–1.36) vs. ICU 0.30 mg × min/L (0.02–1.09) *p* = 0.022) and day 3 (elective 0.86 mg × min/L (0.23–1.43) vs. ICU 0.52 mg × min/L (0.05–2.24), *p* = 0.015), as well as OH/ESOM on day 4 (elective 0.03 mg × min/L (0.02–0.11) vs. ICU 0.05 mg × min/L (0.03–0.18), *p* = 0.009). However, counterintuitively, the metabolite ratios on the iv day (day 4) were not lower than on days of oral administration, as expected (Figure 3). 

On the contrary, between days 1 and 3 only the AUC of OH and DM differed statistically significantly (*p* = 0.010 and *p* = 0.016, respectively). All other differences between days were not statistically significant.

As seen in the table, there was a steady recovery of the mean in vivo activity of CYP3A4 in the cohort of ICU/shock patients, starting from a ratio of S-omeprazole sulfone/S-omeprazole of 0.30, which recovered moderately on day 3 and was 0.70 on day 7 after hospitalization. Unlike intestinal absorption and AUC, hepatic metabolism through CYP3A4 in the elective surgery control cohort appeared not to be significantly compromised after heart surgery.

As indicated by the metabolites 5′-OH-esomeprazole and 5-O-desmethyl esomeprazole, there were no clear effects of shock or cardiac surgery on the enzyme CYP2C19.

**Figure 4:**
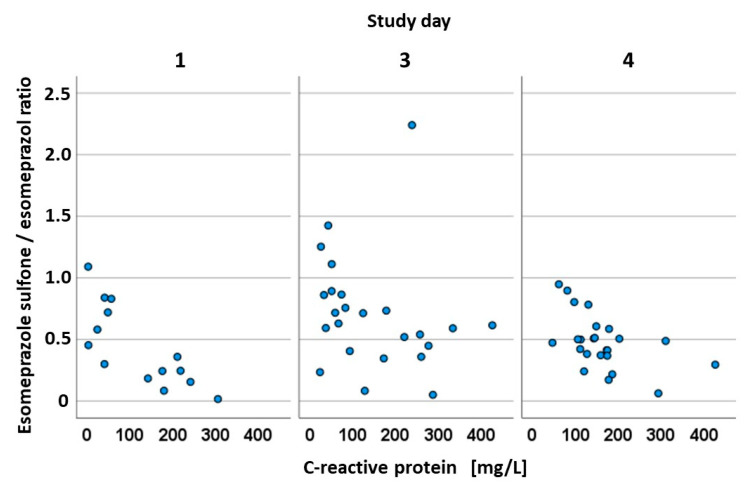
Correlation between esomeprazole–sulfone/esomeprazole ratio and C-reactive protein. Data from both cohorts are combined in this analysis.

The C-reactive protein, a so-called acute phase protein that indicates the severity of an inflammation, which is well-known to increase regularly after surgery and after ischemia, was significantly correlated with the omeprazole sulfone quotient in the first three days investigated (*p* = 0.002, *p* = 0.040, *p* = 0.011, respectively) with a fairly good correlation (r = 0.758, r = 0.422, r = 0.510, respectively) (Figure 4).

**Figure 5:**
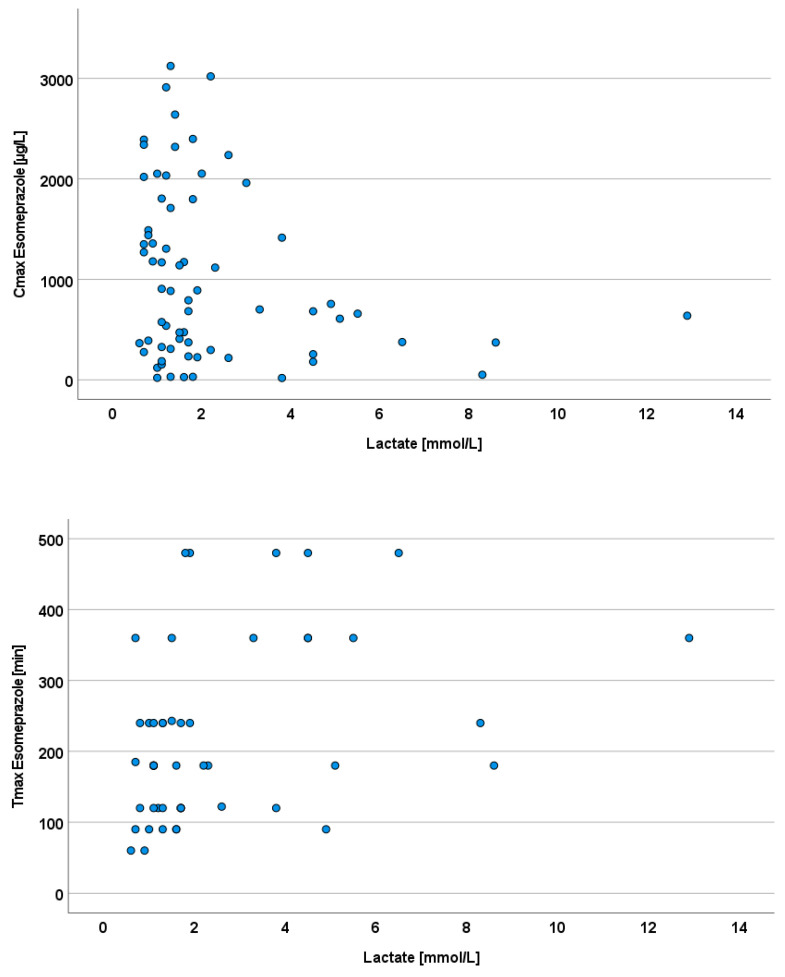
High serum lactate as an indicator of poor enteral drug absorption, as indicated by esomeprazole pharmacokinetics.

Arterial lactate concentrations in the intensive care cohort showed a greater range than in the elective cohort, especially with respect to the maximum values. On the day of admission, 10 out of 11 patients had a pathologically elevated lactate of at least 2 mmol/L, which was defined as the cutoff point for pathologically elevated values. In patients with elevated lactate levels, only a lower Cmax was achieved than in patients with normal-range lactate levels. Arterial lactate on the respective days of pharmacokinetic measurements was negatively correlated with the maximum plasma concentration of esomeprazole (Cmax, Pearson’s r = −0.25, *p* = 0.04) and with the maximum plasma concentration (r = 0.37, *p* = 0.01, Figure 5).

Data on the volume of distribution and total clearance can only be calculated from intravenous dosing. Here, we analyzed the data using pharmacokinetic modeling. The concentration time data after iv dosing was well-described with a one-compartment model. The volume of distribution determined on the day of intravenous administration did not differ between the two groups (elective 17.6 (13.3–36.3) liters in the elective surgery group versus 19.5 (14.2–28.1) liters in the ICU group after cardiogenic shock, *p* = 0.36). Total clearance was 72 (30–130) mL/min in the elective group versus 81 (40–190) mL/min in the ICU group (*p* = 0.17). These volume and clearance data indicate that 4 days after admission, systemic drug distribution and elimination were comparable between both groups, and significant differences may be mainly due to disease- and intervention-related differences in the absorption process.

## 4. Discussion

As an overarching result, we found both impairment and substantial variability in drug absorption even up to 7 days after the insult. CYP3A4-mediated drug metabolism was also significantly compromised in the ICU group in the first two days while recovering from cardiogenic shock. Although some details on pharmacokinetic alterations in critically ill patients can vary depending on the molecular properties of each drug, our data showed that esomeprazole may be a probe drug for enteral absorption and CYP3A4 activity in intensive care medicine. According to Figure 2, drawing at least three blood samples may be advisable, for example, 1, 2, and 4 h after dosing.

A crucial prerequisite behind the design of our study was that within a small time window of a few minutes after application of esomeprazole mups^®^, either by swallowing the tables or by application via the gastric tube as recommended by the pharmaceutical manufacturer, the amount of acid-stable micropellets in the stomach is the same and thus, at almost the same time, there is the same amount of micropellets ready for transport from the stomach into the small intestine. This propulsive transport is then the prerequisite for absorption in the small intestine. That there is a very similar bioavailability of esomeprazole administered through a nasogastric tube compared with oral dosing was also demonstrated earlier in a large number of healthy volunteers [23].

The elective group had higher median maximum esomeprazole concentrations than the ICU cohort, with a peak concentration time twice as high on preoperative days than on postoperative days. This indicates impaired absorption of esomeprazole in critically ill patients when administered enterally. Our finding in this regard is consistent with the results of previous studies in which half of elective patients experienced a 23% decrease in esomeprazole Cmax postoperatively compared with healthy individuals [24]. More than 50% of the patients in our elective surgery cohort had maximum plasma concentrations and AUC values that were so low that a sufficient drug effect could not be anticipated. Interestingly, on day 3, the AUC in the elective surgery group was even lower than that in the critical care cohort on day 3 (Figure 1, Table 3). Reduced gastrointestinal motility has been observed during and after cardiopulmonary bypass [26,27,28]. The use of cardiopulmonary bypass can lead to a pronounced sterile inflammatory reaction in which gastric motility is significantly disturbed immediately after surgery, which could explain the reduced AUC and Cmax values on the postoperative day of the elective cohort. Another proposed explanation is the use of iced cardioplegia during cardiopulmonary bypass surgery, which could cause temporary vagal denervation leading to temporary gastric atony [28]. The low AUC on day 3 could be attributed to a longer retention of the presumably not entirely acid-stable microencapsulated esomeprazole used in this study in the acidic environment of the slow-emptying stomach than to the absorbent mucosa of the small intestine. These results support Berger et al.’s finding that gastral administration reduced AUC, Cmax, and Tmax on the first postoperative day much more than postpyloric administration with opiate administration, causing gastric hypomotility and, consequently, delayed gastric emptying, which has been identified as the most influential factor [9,29]. Furthermore, in our investigation, all patients in the elective cohort received oxycodone analgesic medication at least the first day after surgery. Delayed gastric emptying can cause the enteric coating of esomeprazole microcapsules to be attacked, reducing the efficacy of the acid-sensitive substance. The combination of these findings suggests that in patients who require high doses of opioids after surgery, the intravenous route of administration should be selected for the delivery of the drug to ensure adequate drug levels. 

When the maximal plasma concentrations in the intensive care group on the eighth day of esomeprazole administration are compared with the values in the elective cohort on the first day, it is obvious that several of the patients have already recovered significantly. But even 7 days after shock, the median AUC of esomeprazole was only 145 compared with 271 mg × min/L in the control condition (Table 3). Andersson et al. emphasized the importance of improved patient condition, as well as the effects of numerous doses in this regard [30]. The authors of that study reported a double in Cmax in a group of eight healthy volunteers in 5 days. The investigation by Hasselgren et al. with a cohort of 14 participants similarly found comparable results [24]. The effects were explained by in vitro data showing that both omeprazole and 5-OH-omeprazole could act as reversible and irreversible inhibitors of CYP2C19 and CYP3A4 [31]. Iesu et al. presented evidence of decreased liver function after cardiac arrest with cardiopulmonary resuscitation [32]. They investigated the occurrence of hypoxic hepatitis and acute liver failure after out-of-hospital cardiac arrest. Acute liver failure, defined as hyperbilirubinemia (bilirubin > 1.2 mg/dL) and coagulopathy (INR > 1.5), was identified in 56% of patients three days after ROSC (return of spontaneous circulation). In 7% of cases, hypoxic hepatitis could be diagnosed after only one day. Champigneulle et al. observed comparable results in an observational study in which they evaluated 632 patients with ROSC after an out-of-hospital cardiac arrest over 6 years. Within the first 72 h, 11.4% of them developed hypoxic hepatitis [33]. Restriction of liver function is also described as a possible but understudied consequence after elective cardiac surgery, particularly aortocoronary bypass [34]. Risk factors that existed before surgery, such as right heart failure, low ejection fraction, or NYHA stages II-IV, increase the probability of postoperative liver failure.

Restriction of liver function after cardiac arrest, shock states, or (elective) cardiac surgery can be explained by its high oxygen demand. In particular, cytochrome P450 enzymes rely on a steady supply of oxygen for their reactions, and they are very sensitive to hypoxia being damaged first in the line of drug-metabolizing enzymes. Hypoxic injury to liver cells can result from any type of ischemia, with hypoxic and ischemic ischemia being the most prominent [35,36,37]. Furthermore, procedures such as bypass surgery can trigger a systemic inflammatory response, leading to postoperative liver cell dysfunction [34].

C-reactive protein (CRP) is an inflammatory response parameter induced by interleukin-6, synthesized by the liver, and released into the blood as an acute phase protein that also serves as a hepatic metabolic biomarker [38]. CRP in our ICU population grew steadily from the day of admission to the third study day, i.e., the fourth day after admission. Dell’anna et al. discovered the same phenomenon in their retrospective investigation of 130 patients who had suffered a cardiac arrest. The increase in CRP after ROSC is caused by the so-called post-resuscitation syndrome, which is ultimately a systemic inflammatory response, triggered by ischemic processes during cardiac arrest [39]. A correlation analysis showed a negative correlation between CRP levels and the sulfone–omeprazole quotient on study days 1 through 4, suggesting that increasing CRP levels correlate with decreased CYP3A4 activity. This raises questions about CRP’s role in the altered hepatic metabolism of esomeprazole by CYP enzymes. In a study in 2021, Simon et al. found that high levels of CRP significantly affect the activity of CYP3A4, while the effects were less pronounced on the activity of the CYP2C19 enzyme [37]. This highlights the importance of inflammatory mediators in the activity of the cytochrome P450 system. Dickmann et al. discovered this effect in an in vitro study of hepatocytes exposed to interleukin-6 cytokine [40]. A recent study on patients with severe SARS-CoV-2 infection demonstrated the significant influence of the inflammatory markers IL-6 and CRP on several cytochrome P450 enzymes, including CYP3A and CYP2C19 [41]. In our study, a significant difference was observed in the hydroxy-esomeprazole quotient between the intensive and elective cohorts, where the highest difference was observed on the fourth day of the study in the ICU cohort. On days 1 through 4, the hydroxy quotient was higher than or equal to that of the control group. As a consequence, the CYP2C19 enzyme appears to be less affected by acute disease than the CYP3A4 enzyme. According to Simon et al., lowering CRP concentrations during a hospital stay resulted in a lower down-regulation of CYP enzyme activity, which is why the highest quotients of the three metabolites were calculated on day 4 for the ICU cohort [38].

The enzymes CYP3A4 and CYP2C19 convert esomeprazole to inactive metabolites. CYP3A4 activity is indicated by omeprazole sulfone and the omeprazole sulfone/esomeprazole ratio, while CYP2C19 activity is indicated by 5′-OH-esomeprazole and 5-O-desmethylesomeprazole and their respective ratios to esomeprazole. The pharmacokinetics of the metabolites of esomeprazole did not change significantly postoperatively in the elective cohort. Although the AUC of the three metabolites decreased on day 3, this was well explained by a decrease in bioavailability and AUC, but there were no significant differences in the ratios between the groups; surgery did not influence hepatic metabolic function. The sulfone metabolite had the highest AUC in both research groups [42]. On the first day, we found a substantial difference in omeprazole–sulfone AUC and quotients between elective and ICU populations. The ICU group recovering from cardiac arrest exhibited a median AUC of just one-fifth of that of the elective cohort, while the ratio reached one-third of that of the elective cohort. In the ICU group, the metabolite quotients for omeprazole–sulfone are 0.49 on the iv day, somewhat lower than on day 3 (0.52) and higher than on day 1 (0.30). Due to the first-pass effect of oral delivery, where metabolites have already formed during the first hepatic transit with oral treatment, the concentrations should therefore be higher, which we did not find. However, this unexpected result allows for a more objective statement about the reduced metabolic function of the liver and could also explain why the AUC of esomeprazole in the ICU cohort was not too low. Finally, with less hepatic breakdown, the drug is more active and detectable. On the contrary, the quotient values in the elective cohort follow the predicted patterns. Although the sulfone metabolite quotient in the ICU cohort differed from that of the elective cohort on day 4, the difference was no longer statistically significant. If the sulfone quotient is a reliable indicator of liver function, specifically the activity of the CYP3A4 enzyme, this suggests that it gradually improved during the first eight days after the acute incident.

The median BMI of the ICU cohort was 28.9 kg/m^2^, while the elective cohort was 26.8 kg/m^2^. For the elective cohort, statistical analysis revealed a strong association between body weight and AUC of esomeprazole. These findings may appear to contradict a general principle in pharmacokinetics according to which blood concentrations and parameters, such as Cmax and AUC, are negatively correlated with body weight. However, in both cohorts, a higher BMI was also associated with worse overall health.

This study examined patients aged 41–78 years, with the elective cohort having a median age of 67 years, which is 10 years older than the ICU cohort. This study did not find a correlation between age and pharmacokinetic parameters. However, Andersson et al. found that the rate of metabolization is slightly reduced in the elderly, possibly due to reduced liver perfusion or increased body fat mass [30]. Dose adjustment is not necessary for esomeprazole to have a sufficient effect. Hasselgren et al. suggested an age-related decrease in CYP3A4 activity in older men as the cause of higher AUC values for esomeprazole [24].

In summary, esomeprazole bioavailability was dramatically reduced after cardiac surgery, falling below the values determined in the intensive care cohort on the first day of blood collection. This emphasizes the need to consider drug administration routes and favor intravenous administration in all professions. The condition in healthy adults was mirrored in the control group. The AUC of esomeprazole increased compared with healthy people, perhaps due to changes in metabolic status and reduced liver function caused by acute circulatory shock. The AUC of omeprazole sulfone was significantly reduced in the ICU population, as were Tmax and Cmax. As a result, CYP3A4-mediated omeprazole sulfone production in the liver can be considered considerably degraded. The AUC of the sulfone metabolite in the elective cohort was halved on the postoperative day compared with the preoperative day. Studies have shown that omeprazole has a higher bioavailability in patients with liver cirrhosis, with a bioavailability of 98% [43]. This increased bioavailability is likely due to a reduction in first-pass metabolism and a prolonged elimination half-life.

### Limitations

An important point to consider when utilizing esomeprazole as a probe drug for drug absorption is the fact that blood concentrations (Cmax) may increase by a factor of 1.5 to 2 after multiple doses when compared with the first dose [42]. In that publication, the AUC even increased by a factor of 2.6. Those effects are most likely caused by self-inhibition of biotransformation. In our pharmacokinetic data and metabolic ratios (Table 4), no major effects of self-inhibition were seen, which may be explained by the opposite effects of that inhibition and generally improving health.

Food consumption after esomeprazole treatment was not routinely controlled, which could have decreased absorption. However, that corresponds to clinical requirements and typical clinical practice. As shown in Table 2, major comedications administered to both groups (Table 2) do not have a major interaction potential, but comedications given only to a few patients could also not be included in the analysis for statistical reasons. In clinical settings, coordinated medical treatments and maintaining a suitable oral diet are equally crucial to ICU care, which influences study results. The gender distribution of the patients was both a strength and a weakness, with the majority of male patients providing good interindividual comparability while also making generalization to female genders problematic. 

We did not investigate genetic variations that cause slow or deficient CYP2C19 metabolism in individual patients [44,45,46]. However, in our sample, there was no outlier with extremely low formation of hydroxylated and demethylated esomeprazole metabolites formed by CYP2C19, which is reasonable considering a population frequency of only 3% in the European population. Furthermore, the effects of the CYP2C19 genotype appear to be less in elderly people, such as our population, compared with young people [47]. And, as seen by the about 10-fold lower ratios of 5-hydroxy omeprazole to omeprazole sulfone (Table 4), polymorphic CYP2C19 may indeed account only for about 10 to 20% of esomeprazole elimination.

As seen in the figures showing the single subject data, there were a few unexplained outliers. Since errors explaining those outliers were not found, we did not exclude these datapoints. However, all data were analyzed using nonparametric statistics; thus, data in the tables and the validity of the conclusions was not affected by the outliers.

## 5. Conclusions

From our study, it becomes obvious that gastrointestinal drug absorption is significantly reduced in critically ill cardiac patients, even more so than after major elective (cardiac) surgery. Based on our findings, we suggest that medications should preferably be administered intravenously after a comparable (elective) surgical intervention, as well as during critical illness in general, to achieve suitably high drug levels. Furthermore, after the acute event, liver function, evaluated by esomeprazole hepatic metabolism, is impaired in critically ill patients. The CYP3A4 enzyme, which is inhibited in its activity by a variety of factors, appears to represent a particularly exposed factor in this context due to both enzyme inhibition by multiple comedications and down-regulation by inflammatory cytokines.

## Figures and Tables

**Table 1:** Demographics and baseline characteristics (median (min–max)), Abbreviations: eGFR = estimated glomerular filtration rate; ALT = alanine aminotransferase; AST = aspartate aminotransferase; GGT = gamma-glutamyltransferase; AP = alkaline phosphatase; CK = creatinine kinase; LDH = lactate dehydrogenase.

	Normal Range	Elective/Control Cohort	ICU/Shock Cohort	U-Test
Gender (f:m)	-	2:9	0:14	
Age (years)	-	67 (46–76)	68.5 (68–69)	0.134
Height (cm)	-	178 (163–190)	185 (180–190)	0.066
Body weight (kg)	-	94 (65–122)	105 (100–110)	0.467
Body mass index (kg/m^2^)	18.5–25	32.4 (24.5–40.5)	30.9 (27.7–34.0)	0.809
Body surface area (m^2^)	-	2.05 (1.70–2.48)	2.29 (2.28–2.29)	0.183
Serum creatinine [mg/dL]	0.5–1.2	0.9 (0.77–1.51)	1.51 (1.28–1.74)	**0.011**
eGFR [mL/min]	>60	60 (45.8–60)	47.85 (40–55.7)	0.167
Bilirubin [mg/dL]	≤1.2	0.6 (0.4–1.4)	0.55 (0.4–0.7)	0.652
ALT [U/L]	≤45	35 (26–75)	157.5 (117–198)	**<0.001**
AST [U/L]	≤35	29 (24–44)	150 (129–171)	**<0.001**
GGT [U/L]	12–64	42 (29–195)	73.5 (39–108)	0.3
AP [U/L]	40–150	74 (39–138)	91 (88–94)	0.608
CRP [mg/L]	≤5.0	2.4 (0.4–17.1)	2.3 (1.2–3.4)	**0.002**
CK [U/L]	30–200	93 (36–162)	517 (484–550)	**<0.001**
LDH [U/L]	≤248	255 (213–441)	568.5 (498–639)	**0.004**
Troponin-I [ng/L]	≤14	16.5 (3.2–651)	18,939.5 (15,126–22,753)	**0.003**
Lactate [mmol/L]	≤1.8	-	5.15 (5.0–5.3)	n/a

**Table 2:** Comedication of the respective groups.

Medication	Elective/Control Cohort (*n* (*%*))	ICU/Study Cohort (*n* (*%*))
Acetyl salicylic acid	9 (81.8)	10 (71.4)
Bisoprolol	6 (54.6)	7 (50)
Cefazoline	9 (81.8)	-
Clonidine	-	9 (64.3)
Dobutamine	7 (63.6)	-
Furosemide	9 (81.8)	11 (78.6)
Heparin (unfractionated)	10 (90.9)	12 (85.7)
Insulin	8 (72.7)	11 (78.6)
Ipratropium bromide	-	10 (71.4)
Isoflurane	-	10 (71.4)
Lorazepam	-	5 (35.7)
Metamizole	6 (54.6)	6 (42.9)
Noradrenaline	10 (90.9)	14 (100)
Oxycodone	11 (100)	-
Paracetamol	11 (100)	-
Piperacillin/Tazobactam	-	11 (78.6)
Potassium chloride	10 (90.9)	10 (71.4)
Prasugrel	-	5 (35.7)
Propofol	11 (100)	12 (85.7)
Ramipril	7 (63.6)	6 (42.9)
Statin (any)	9 (81.8)	7 (50)
Sufentanil	-	14 (100)
Torasemide	5 (45.5)	-
Vancomycin	-	6 (42.9)

The esomeprazole peak plasma concentrations (Cmax), times of peak plasma concentrations (Tmax), and area under the concentration–time curve (AUC) were all subject to high variability between subjects. As illustrated in Figure 1, there was also significant intraindividual variation between days.

**Table 3:** ^#^ Significance according to the Mann–Whitney U test; ^##^ compared to day 1 of the control group, NA not applicable, the column with iv dose is marked in gray to indicate that no intestinal absorption or first-pass metabolism occurs. Due to its eight concentrations taken at different time points, AUC is the most precise parameter. This parameter shows that intestinal absorption in the ICU/shock cohort was on average reduced by about 40%, even after 7 days of treatment, compared to the control group (measurements before surgery). Data for the time to maximum blood concentrations and maximum blood concentrations corresponded to those showing significantly delayed and quantitatively reduced absorption, particularly on day 1 in the ICU group. In several patients in the ICU group, this delayed absorption was found even on day 7 of hospitalization.

Group	Day 1 Enteral	Day 3 Enteral	Day 4 Intravenous	Day 7 Enteral
Esomeprazole maximum plasma concentration (Cmax) [µg/L]				
ICU/after shock	474 (19–891)	474 (21–1711)	2020 (1306–3124)	831 (153–1440)
Control group	1106 (347–1775)	298 (28–756)	2563 (1140–4178)	
*p* ^#^	0.001	0.055	0.072	0.088 ^##^
Time of esomeprazole maximum plasma concentration (Tmax) [min]				
ICU/after shock	242 (120–480)	122 (60–360)	NA	180 (60–480)
Control group	120 (60–360)	240 (90–480)		
*p*	0.005	0.055		0.36 ^##^
Esomeprazole area under the concentration time curve (AUC) [mg × min/L]				
ICU/after shock	137 (4.5–269)	125 (5–520)	397 (182–589)	145 (31.2–275)
Control group	271 (85.5–384)	97 (4.6–206)	482 (250–753)	
*p*	0.005	0.072	0.19	

**Table 4:** ^#^ Significance according to the Mann–Whitney U test; ^##^ compared to day 1 of the control group, NA not applicable, the column with iv dose is marked in gray to indicate that no further intestinal absorption nor first-pass metabolism takes place. Please note that metabolic ratios after intravenous dosing are not comparable to those after oral dosing because only in oral dosing there is first-pass metabolism in the intestinal epithelia and in the liver. The AUCs of the metabolites are not given here because they reflect both absorption and metabolism.

Group	Day 1	Day 3	Day 4	Day 7
5′-OH-esomeprazole AUC [mg × min/L]				
ICU/shock	10.5 (1.4–35.2)	15.9 (1.4–44.8)	23.3 (10.1–62.9)	12.2 (6.0–37)
Control	12.4 (5.4–57.2)	5.27 (0.72–13.9)	13.6 (8.8–23.6)	
*p* ^#^	0.936	0.063	0.119	0.699
5-O-Desmethyl esomeprazole AUC [mg × min/L]				
ICU/shock	1.14 (0.08–4.1)	0.63 (0.03–3.54)	1.5 (0.6–4.9)	1.2 (0.2–2.8)
Control	1.4 (0.9–5.1)	0.45 (0.04–2.24)	1.61 (0.5–3.5)	
*p* ^#^	0.202	0.361	0.776	0.133
Esomeprazole sulfone AUC [mg × min/L]				
ICU/shock	41.0 (5.1–287)	70.3 (4.9–303)	155 (25.8–331)	101 (3.4–226)
Control	198 (45.5–379)	95.8 (1.2–247)	217 (108–397)	
*p* ^#^	0.001	0.955	0.167	0.007
5′-OH-esomeprazole/esomeprazole AUC ratio (reflecting CYP2C19)				
ICU/shock	0.08 (0.03–0.74)	0.06 (0.02–0.22)	0.05 (0.03–0.18)	0.11 (0.05–0.40)
Control	0.05 (0.02–0.13)	0.06 (0.03–0.35)	0.03 (0.02–0.11)	
*p* ^#^	0.119	0.776	0.009	0.034 ^##^
5-O-Desmethyl esomeprazole/esomeprazole AUC ratio (reflecting CYP2C19)				
ICU/shock	0.01 (0.00–0.06)	0.00 (0.00–0.02)	0.00 (0.00–0.01)	0.01 (0.00–0.05)
Control	0.01 (0.00–0.05)	0.01 (0.00–0.04)	0.00 (0.00–0.01)	
*p* ^#^	0.865	0.072	0.167	0.748 ^##^
Esomeprazole sulfone/esomeprazole AUC ratio (reflecting CYP3A4)				
ICU/shock	0.30 (0.02–1.09)	0.52 (0.05–2.24)	0.49 (0.06–0.95)	0.70 (0.11–1.08)
Control	0.79 (0.35–136)	0.86 (0.23–1.43)	0.42 (0.24–0.80)	
*p* ^#^	0.022	0.015	0.733	0.401 ^#^

## Data Availability

Data are available from the authors upon reasonable request.

## References

[1] Elke G., van Zanten A.R., Lemieux M., McCall M., Jeejeebhoy K.N., Kott M., Jiang X., Day A.G., Heyland D.K. (2016). Enteral versus parenteral nutrition in critically ill patients: An updated systematic review and meta-analysis of randomized controlled trials. Crit. Care.

[2] Boucher B.A., Wood G.C., Swanson J.M. (2006). Pharmacokinetic changes in critical illness. Crit. Care Clin..

[3] Morales Castro D., Dresser L., Granton J., Fan E. (2023). Pharmacokinetic Alterations Associated with Critical Illness. Clin. Pharmacokinet..

[4] Zaloga G.P., Roberts P.R., Marik P. (2003). Feeding the hemodynamically unstable patient: A critical evaluation of the evidence. Nutr. Clin. Pract..

[5] McClave S.A., Chang W.K. (2003). Feeding the hypotensive patient: Does enteral feeding precipitate or protect against ischemic bowel?. Nutr. Clin. Pract..

[6] Jabbar A., Chang W.K., Dryden G.W., McClave S.A. (2003). Gut immunology and the differential response to feeding and starvation. Nutr. Clin. Pract..

[7] Fruhwald S., Holzer P., Metzler H. (2007). Intestinal motility disturbances in intensive care patients pathogenesis and clinical impact. Intensive Care Med..

[8] Cohn S.M., Sawyer M.D., Burns G.A., Tolomeo C., Milner K.A. (1996). Enteric absorption of ciprofloxacin during tube feeding in the critically ill. J. Antimicrob. Chemother..

[9] Berger M.M., Berger-Gryllaki M., Wiesel P.H., Revelly J.P., Hurni M., Cayeux C., Tappy L., Chiolero R. (2000). Intestinal absorption in patients after cardiac surgery. Crit. Care Med..

[10] Heyland D.K., Tougas G., King D., Cook D.J. (1996). Impaired gastric emptying in mechanically ventilated, critically ill patients. Intensive Care Med..

[11] Southren D.L., Nardone A.D., Haastrup A.A., Roberts R.J., Chang M.G., Bittner E.A. (2021). An examination of gastrointestinal absorption using the acetaminophen absorption test in critically ill patients with COVID-19: A retrospective cohort study. Nutr. Clin. Pract..

[12] Chiolero R.L., Revelly J.P., Berger M.M., Cayeux M.C., Schneiter P., Tappy L. (2003). Labeled acetate to assess intestinal absorption in critically ill patients. Crit. Care Med..

[13] Soppi V., Kokki H., Koivisto T., Lehtonen M., Helin-Tanninen M., Lehtola S., Rinne J. (2007). Early-phase pharmacokinetics of enteral and parenteral nimodipine in patients with acute subarachnoid haemorrhage—A pilot study. Eur. J. Clin. Pharmacol..

[14] Abboud T., Andresen H., Koeppen J., Czorlich P., Duehrsen L., Stenzig J., Westphal M., Regelsberger J. (2015). Serum levels of nimodipine in enteral and parenteral administration in patients with aneurysmal subarachnoid hemorrhage. Acta Neurochir..

[15] Smith B.S., Yogaratnam D., Levasseur-Franklin K.E., Forni A., Fong J. (2012). Introduction to drug pharmacokinetics in the critically ill patient. Chest.

[16] Calabuig R., Seggerman R.E., Weems W.A., Weisbrodt N.W., Moody F.G. (1990). Gallbladder and gastrointestinal motility after hemorrhagic shock. Surgery.

[17] Moody F.G., Calabuig R., Li Y.F., Harari Y., Rodriguez L.F., Weisbrodt N.W. (1990). Biliary and gut function following shock. J. Trauma.

[18] Kalima T.V., Kivilaakso E., Lempinen M. (1981). Disturbances in gastric motility during hypovolaemic shock. Scand. J. Gastroenterol. Suppl..

[19] Glatzle J., Leutenegger C.M., Mueller M.H., Kreis M.E., Raybould H.E., Zittel T.T. (2004). Mesenteric lymph collected during peritonitis or sepsis potently inhibits gastric motility in rats. J. Gastrointest. Surg..

[20] Forsberg J., Bedard E., Mahmoud S.H. (2023). Bioavailability of Orally Administered Drugs in Critically Ill Patients. J. Pharm. Pract..

[21] Shin J.M., Kim N. (2013). Pharmacokinetics and pharmacodynamics of the proton pump inhibitors. J. Neurogastroenterol. Motil..

[22] Vahdatpour C., Collins D., Goldberg S. (2019). Cardiogenic Shock. J. Am. Heart Assoc..

[23] Sostek M.B., Chen Y., Skammer W., Winter H., Zhao J., Andersson T. (2003). Esomeprazole administered through a nasogastric tube provides bioavailability similar to oral dosing. Aliment. Pharmacol. Ther..

[24] Hasselgren G., Hassan-Alin M., Andersson T., Claar-Nilsson C., Röhss K. (2001). Pharmacokinetic Study of Esomeprazole in the Elderly. Clin. Pharmacokinet..

[25] Xu Y., Tian X., Wang W., Tian W., Zhang T., Sun J., Zhou Q., Shao C. (2021). Pharmacokinetics of Esomeprazole in Critically Ill Patients. Front. Med..

[26] Lin Y., Chen M., Peng Y., Chen Q., Li S., Chen L. (2021). Feeding intolerance and risk of poor outcome in patients undergoing cardiopulmonary bypass surgery. Br. J. Nutr..

[27] Gu Y.J., de Kroon T.L., Elstrodt J.M., Rakhorst G. (2006). Gastrointestinal motility during cardiopulmonary bypass: A sonomicrometric study. Artif. Organs.

[28] Goldhill D.R., Whelpton R., Winyard J.A., Wilkinson K.A. (1995). Gastric emptying in patients the day after cardiac surgery. Anaesthesia.

[29] Dive A., Moulart M., Jonard P., Jamart J., Mahieu P. (1994). Gastroduodenal motility in mechanically ventilated critically ill patients: A manometric study. Crit. Care Med..

[30] Andersson T., Hassan-Alin M., Hasselgren G., Röhss K., Weidolf L. (2001). Pharmacokinetic studies with esomeprazole, the (S)-isomer of omeprazole. Clin. Pharmacokinet..

[31] Shirasaka Y., Sager J.E., Lutz J.D., Davis C., Isoherranen N. (2013). Inhibition of CYP_2_C_19_ and CYP_3_A_4_ by omeprazole metabolites and their contribution to drug-drug interactions. Drug Metab. Dispos..

[32] Iesu E., Franchi F., Zama Cavicchi F., Pozzebon S., Fontana V., Mendoza M., Nobile L., Scolletta S., Vincent J.L., Creteur J. (2018). Acute liver dysfunction after cardiac arrest. PLoS ONE.

[33] Champigneulle B., Geri G., Bougouin W., Dumas F., Arnaout M., Zafrani L., Pène F., Charpentier J., Mira J.P., Cariou A. (2016). Hypoxic hepatitis after out-of-hospital cardiac arrest: Incidence, determinants and prognosis. Resuscitation.

[34] Chacon M.M., Schulte T.E. (2018). Liver Dysfunction in Cardiac Surgery—What Causes It and Is There Anything We Can Do?. J. Cardiothorac. Vasc. Anesth..

[35] Ebert E.C. (2006). Hypoxic liver injury. Mayo Clin. Proc..

[36] Fuhrmann V., Kneidinger N., Herkner H., Heinz G., Nikfardjam M., Bojic A., Schellongowski P., Angermayr B., Schoniger-Hekele M., Madl C. (2011). Impact of hypoxic hepatitis on mortality in the intensive care unit. Intensive Care Med..

[37] Fuhrmann V., Kneidinger N., Herkner H., Heinz G., Nikfardjam M., Bojic A., Schellongowski P., Angermayr B., Kitzberger R., Warszawska J. (2009). Hypoxic hepatitis: Underlying conditions and risk factors for mortality in critically ill patients. Intensive Care Med..

[38] Simon F., Gautier-Veyret E., Truffot A., Chenel M., Payen L., Stanke-Labesque F., Tod M. (2021). Modeling Approach to Predict the Impact of Inflammation on the Pharmacokinetics of CYP_2_C_19_ and CYP_3_A_4_ Substrates. Pharm. Res..

[39] Dell’anna A.M., Bini Viotti J., Beumier M., Orbegozo-Cortes D., Donadello K., Scolletta S., Vincent J.L., Taccone F.S. (2014). C-reactive protein levels after cardiac arrest in patients treated with therapeutic hypothermia. Resuscitation.

[40] Dickmann L.J., Patel S.K., Rock D.A., Wienkers L.C., Slatter J.G. (2011). Effects of interleukin-6 (IL-6) and an anti-IL-6 monoclonal antibody on drug-metabolizing enzymes in human hepatocyte culture. Drug Metab. Dispos..

[41] Lenoir C., Terrier J., Gloor Y., Curtin F., Rollason V., Desmeules J.A., Daali Y., Reny J.L., Samer C.F. (2021). Impact of SARS-CoV-2 Infection (COVID-19) on Cytochromes P450 Activity Assessed by the Geneva Cocktail. Clin. Pharmacol. Ther..

[42] Hassan-Alin M., Andersson T., Bredberg E., Röhss K. (2000). Pharmacokinetics of esomeprazole after oral and intravenous administration of single and repeated doses to healthy subjects. Eur. J. Clin. Pharmacol..

[43] Andersson T., Olsson R., Regårdh C.G., Skånberg I. (1993). Pharmacokinetics of [^14^C]omeprazole in patients with liver cirrhosis. Clin. Pharmacokinet..

[44] Seeringer A., Kirchheiner J. (2008). CYP_2_D_6_-, CYP_2_C_9_- and CYP_2_C_19_-based dose adjustments: When do they make sense?. Der Internist.

[45] Brockmöller J., Kirchheiner J., Meisel C., Roots I. (2000). Pharmacogenetic diagnostics of cytochrome P450 polymorphisms in clinical drug development and in drug treatment. Pharmacogenomics.

[46] Rost K.L., Brockmöller J., Esdorn F., Roots I. (1995). Phenocopies of poor metabolizers of omeprazole caused by liver disease and drug treatment. J. Hepatol..

[47] Ducker C.M., Brockmoller J. (2019). Genomic Variation and Pharmacokinetics in Old Age: A Quantitative Review of Age- vs. Genotype-Related Differences. Clin. Pharmacol. Ther..

